# Overexpression of miR-532-5p restrains oxidative stress response of chondrocytes in nontraumatic osteonecrosis of the femoral head by inhibiting ABL1

**DOI:** 10.1515/med-2024-0943

**Published:** 2024-03-30

**Authors:** Peng Shang, Ying Liu, Jie Ren, Qingqing Liu, Haobo Song, Junqing Jia, Qiang Liu

**Affiliations:** Department of Orthopedics, Shanxi Bethune Hospital, Shanxi Academy of Medical Sciences, Tongji Shanxi Hospital, Third Hospital of Shanxi Medical University, Shanxi, 030032, P.R. China; Tongji Hospital, Tongji Medical College, Huazhong University of Science and Technology, Wuhan, Hubei, 430030, P.R. China; Department of Oncology, Second Hospital of Shanxi Medial University, Taiyuan, Shanxi, 030001, P.R. China; Department of Orthopedics, Shanxi Bethune Hospital, Shanxi Academy of Medical Sciences, Tongji Shanxi Hospital, Third Hospital of Shanxi Medical University, No. 99, Longcheng Street, Taiyuan, Shanxi, 030032, P.R. China; Tongji Hospital, Tongji Medical College, Huazhong University of Science and Technology, No. 1095, Jiefang Avenue, Wuhan, Hubei, 430030, P.R. China

**Keywords:** nontraumatic osteonecrosis of the femoral head, serology, oxidative stress, miR-532-5p, Abelson tyrosine-protein kinase 1, chondrocyte

## Abstract

This study is to probe into the meaning of serum miR-532-5p in nontraumatic osteonecrosis of the femoral head (ONFH), and a molecular mechanism of miR-532-5p in the development of nontraumatic ONFH. This study enrolled 96 patients diagnosed with nontraumatic ONFH and 96 patients with femoral neck fracture. The levels of miR-532-5p, ABL1, MMP-3, MMP-13, and cleaved-caspase3 were determined. Radiographic progression was assessed by ARCO staging system. Visual analog scale (VAS) and Harris hip score (HHS) were employed for evaluation of the symptomatic severity of nontraumatic ONFH. Cell viability and apoptosis in chondrocytes isolated from clinical samples were investigated with CCK-8 and flow cytometry. The levels of lactic dehydrogenase (LDH), superoxide dismutase (SOD), and malondialdehyde (MDA), mitochondrial membrane potential (Δ*Ψ*m), and reactive oxygen species (ROS) were determined. miR-532-5p was downregulated in tissues and serum of patients with nontraumatic ONFH, negatively related with ARCO staging and VAS, and positively correlated with HHS. Cell apoptosis, LDH, MDA, and ROS strengthened, while cell viability, Δ*Ψ*m, and SOD reduced in chondrocytes of nontraumatic ONFH patients. ABL1 was upregulated in cartilage tissues from nontraumatic ONFH patients. miR-532-5p targeted ABL1, and overexpressed miR-532-5p alleviated nontraumatic ONFH-induced oxidative stress damage of chondrocytes by restraining ABL1. miR-532-5p ameliorated oxidative stress injury in nontraumatic ONFH by inhibiting ABL1.

## 1 Introduction

Osteonecrosis of the femoral head (ONFH) refers to a disabling clinical disease of the hip which conduces to severe pain or joint disability [1], including traumatic and nontraumatic causes [2]. Reportedly, glucocorticoids (GC) and alcohol consumption have been recognized as leading risk factors for nontraumatic ONFH [3,4]. Treatment options for ONFH include non-surgical treatment and surgical treatment, and core decompression is currently the most commonly used treatment for ONFH in its early stages [5]. If left untreated, ONFH may progress to collapse and secondary arthritis [6]. At present, the specific pathological mechanism of ONFH remains ill-defined, and clinicians are faced with clinical challenges in developing targeted treatments for nontraumatic osteonecrosis [7].

Articular cartilage consists of a small number of specific cells, the chondrocytes and dense extracellular matrix distributed between the chondrocytes [8]. Degeneration of articular cartilage was reported to increase instability of the hip joint and expedite the development of ONFH [9], suggesting that prevention and early treatment of cartilage damage may contribute to ameliorating the progression of ONFH. Of note, the disorder of bone homeostasis is thought to be primarily caused by oxidative stress [10] which acts as a critical cause of articular cartilage degradation [11]. Since the pathogenesis of nontraumatic ONFH is intimately correlated to oxidative stress [12], how to reduce oxidative stress-induced injury in articular cartilage of nontraumatic ONFH patients is of great significance for the treatment of the disease.

Abelson tyrosine-protein kinase 1 (ABL1) belongs to the ABL family of tyrosine kinases which has been reported to promote tumor progression and metastasis in a variety of solid tumors [13]. There was convincing evidence indicating that reactive oxygen species (ROS) activated ABL1 to accelerate disease progression [14]. Based on data analysis, a total of ten oxidative stress-related hub genes were found to be differentially expressed in ONFH in which ABL1 was significantly upregulated [15]. In contrast to other genes, the role of ABL1 in ONFH is unclear.

MicroRNAs (miRs) feature in multiple physiological and pathophysiological processes as important regulators, and play pivotal roles in biological processes including cell proliferation, differentiation, apoptosis, and carcinogenesis [16]. Deregulation of miRNAs has been revealed to be implicated in the pathogenesis of ONFH [17]. Efforts have been made to identify serum biomarkers for the early detection of ONFH [18]. For instance, serum miR-93-5p and miR-320a were demonstrated to be overexpressed in patients with ONFH and hold great promise for the early diagnosis and treatment of traumatic ONFH [19]. Previously, downregulated miR-532-5p contributed to the progression of bone remodeling by acting as an intermediate between parathyroid hormone and matrix metalloproteinase (MMP)-13 [20]. Nevertheless, research concerning the impact of miR-523-5p in oxidative stress response and the significance of serum miR-532-5p in nontraumatic ONFH remains insufficient. Herein, our bioinformatics prediction revealed binding of miR-532-5p to ABL1. Consequently, we hypothesized that miR-532-5p could affect the protein expression of ABL1 by targeting ABL1, thereby affecting the proliferation and apoptosis of chondrocytes, with the intention to explore the relationship between miR-532-5p and ABL1, and the role of miR-532-5p in oxidative stress response and its diagnostic value in nontraumatic ONFH.

## 2 Materials and methods

### 2.1 Clinical samples

A total of 96 serum samples from patients with nontraumatic ONFH and corresponding femoral head cartilage tissue samples after total hip arthroplasty were collected at the Orthopaedics Department of Tongji Hospital from October 2020 to July 2021. The diagnosis of nontraumatic ONFH was confirmed by X-radiography, MRI, and CT. The inclusion criteria for patients with steroid-induced ONFH were an average daily dose of ≥16.6 mg or a highest daily dose of 80 mg of prednisolone equivalent within 1 year prior to the presence of symptoms or radiological presentation. For the inclusion of alcohol-induced ONFH, patients had a history of alcohol intake >400 mL/week for over 6 months (any type of alcoholic beverage) and were diagnosed within 1 year after alcohol intake, and they should suffer from no other risk factors. In the meanwhile, 96 sex- and age-matched femoral neck fracture (FNF) patients without ONFH and osteoarthritis were categorized into the control group. Patients were excluded if they had primary autoimmune diseases including osteoarthritis, spinal dissolution, spondylolysis, rheumatoid arthritis, systemic lupus erythematosus or other autoimmune diseases, or histories of tumors.

Fasting venous blood was collected at 8:00 AM from all participants after fasting overnight. Blood samples were collected in vacuum blood collection tubes lacking heparin and then centrifuged at 3,000*g* for 10 min at 4°C to separate the serum. Next, the supernatant was divided into 100 µL aliquots and stored at −80°C until processing. The oxidative stress indicators including lactic dehydrogenase (LDH), superoxide dismutase (SOD), and malondialdehyde (MDA) in serum of nontraumatic ONFH patients and FNF controls were blindly assessed by an enzyme-linked immunosorbent assay (ELISA) kit. Quantitative reverse transcription-polymerase chain reaction (qRT-PCR) assay was applied to determine the expression level of miR-532-5p in serum.

### 2.2 Definition of radiographic progression

Radiographic severity was defined by Association Research Circulation Osseous (ARCO) staging system [21]. Stage I: normal X-ray but positive MRI or bone scan; stage II: abnormal X-ray (subtle symptoms of focal osteoporosis, osteosclerosis, or cysts in the femoral head); stage III: X-ray or CT scan shows fracture or depression in the subchondral or necrotic area (stage III is further separated into stage IIIA [early stage, depression in the femoral head ≤2 mm] and IIIB [late stage, depression in the femoral head >2 mm]); stage IV: X-ray osteoarthritis is associated with joint space narrowing, acetabulum changes, and/or joint destruction. This study included ONFH patients with ARCO ≥ stage II, and the radiographic results were read and evaluated by two experienced radiologists who were blind to the experimentation (the relevant information of the two radiologists is shown in the supplementary files). The *Kappa* value was employed to assess the result consistency, and *Kappa* value ≥0.8 was considered significant.

### 2.3 Determination of symptomatic severity

Visual analog scale (VAS) and Harris hip score (HHS) were utilized to assess the symptomatic severity of nontraumatic ONFH. For VAS, patients were asked to select a score of 0 to 10 according to their own feelings to quantify the degree of pain: 0, no pain and 10, extreme pain. HHS was employed for measurement of overall pain, range of motion (ROM), function, and deformity. The severity of pain and the need for pain medication were measured by pain items. Functional items were divided into daily activities and gait. The deformity items were implemented to survey internal rotation, hip flexion, adduction, and the differences of extremity length, and hip ROM was examined by ROM domain. The HHS was ranked from 0 (worst functional outcome and maximum pain) to 100 (best functional outcome and minimum pain). The explanation of results using HHS was as follows: <70 (poor result: patients had poor hip function, significant or very painful pain, poor mobility or bed rest, and moderate or severe claudication), 70–79 (fair result: patients had poor hip function, significant or mild pain, poor mobility, and mild claudication), 80–89 (good result: patients had good hip function, mild or sometimes painful pain, good mobility, and a basically normal gait), and >90 (excellent result: patients had good hip function with little or no pain, good mobility, and a normal gait).

### 2.4 ELISA

LDH, SOD, and MDA levels in serum from patients and chondrocytes were assessed by the corresponding kits including LDH kit (J20930), SOD (J21118), and MDA (J20465), and all kits were obtained from GILED Biotechnology Co., Ltd (Wuhan, China). To be specific, the samples to be tested were added to a microplate and wrapped overnight, followed by PBS washing (3 × 5 min) and 1 h blocking with 5% bovine serum albumin (BSA) (100 μL/well). Afterward, PBS buffer (with 5% BSA) was used to dilute corresponding primary antibodies, and the antibodies underwent 3 h incubation onto the 96-well plate (100 μL/well), prior to PBS washing for three times, 5 min each. HRP-conjugated secondary antibodies were seeded onto the 96-well plate at a density of 100 μL per well for 1 h incubation, followed by PBS washing. Successively, the plate was added with 10 μL of substrate and placed for 10–15 min at 37°C. The absorbance value of each sample was measured at 450 nm.

### 2.5 Isolation and culture of primary chondrocytes

Human cartilage samples were obtained from patients with nontraumatic ONFH and patients with FNF who underwent total hip arthroplasty. The primary articular chondrocytes were extracted from articular cartilage by enzymatic digestion [22]. In brief, cartilage tissues were washed with 1% PBS and chopped into approximately 1 mm^3^ pieces, followed by digestion with 0.25% trypsinase (CAS: 9002-07-7, Beijing Solarbio Science & Technology Co., Ltd, Beijing, China) and 0.2% Type Ⅱ collagenase (CAS: 9001-12-1, MX1002, Shanghai Maokang Biotechnology Co, Ltd, Shanghai, China). Next, the isolated chondrocytes were inoculated as monolayers and cultured in the DMEM/F12 medium (SNM-004E, Sunncell Biotech Co., Ltd, Wuhan, China) supplemented with 10% fetal bovine serum plus 0.1% penicillin and streptomycin. The third generation of primary chondrocytes and supernatant were collected for further analysis.

### 2.6 Cell transfection

After 0.25% trypsinization, chondrocytes were seeded onto 96-well plates. Upon 60–70% confluence, the cells underwent transfection with miR-532-5p mimic, OE-ABL1, and their negative control (mimic-NC and OE-NC) (GenePharma, Shanghai, China) plasmids using a Lipofectamine^TM^ 3000 kit (Invitrogen, Carlsbad, CA, USA) at a concentration of 75 nM. The cells were divided into control, mimic-NC, miR-532-5p mimic, mimic-NC + OE-NC, miR-532-5p mimic + OE-NC, mimic-NC + OE-ABL1, and miR-532-5p mimic + OE-ABL1 groups.

### 2.7 qRT-PCR

Following extraction of total RNA using TRIZOL (15596026; Invitrogen, Carlsbad, CA, USA) a PrimeScript RT reagent kit (TaKaRa, Tokyo, Japan) was employed for RT. Gene expression was determined using LightCycler 480 (Roche, Indianapolis, IN, USA) according to the instruction of SYBR Green Mix (Roche). GAPDH and U6 served as the internal controls, and data analysis was performed using the 2^–ΔΔCt^ method. The sequences from Sangon (Shanghai, China) are described in Table 1.

**Table 1:** Primer sequences

Name of primer	Sequences
miR-532-5p-F	5′-GCGCGCATGCCTTGAGTGTAG-3′
miR-532-5p-R	5′-ATCCAGTGCAGGGTCCGAGG-3′
U6-F	5′-CTCGCTTCGGCAGCACA-3′
U6-R	5′-AACGCTTCACGAATTTGCGT-3′
ABL1-F	5′-AATCCAAGAAGGGGCTGTCC-3′
ABL1-R	5′-TGGTTTGGGCTTCACACCAT-3
GAPDH-F	5′-AATGGGCAGCCGTTAGGAAA-3′
GAPDH-R	5′-GCGCCCAATACGACCAAATC-3′

Notes: F, forward; R, reverse.

### 2.8 Western blotting

Total protein was extracted from tissues or cells, followed by protein concentration assessment by a BCA kit (P0012S, Beyotime, Shanghai, China). Proteins (50 μg) were dissolved in 2× sodium dodecyl sulfate (SDS) loading buffer, then boiled (100°C for 5 min), separated by SDS-polyacrylamide gel electrophoresis, and then transferred onto polyvinylidene fluoride membranes. Subsequent to blocking in 5% skim milk powder, diluted primary antibodies of ABL1 (ab15130, 1:1,000; Abcam, Cambridge, MA, USA), MMP-3 (ab52915, 1:1,000; Abcam), MMP-13 (ab39012, 1:3,000; Abcam), cleaved-caspase3 (ab32042, 1:500; Abcam), and GAPDH (ab181602, 1:10,000; Abcam) were added for incubation with the membranes at 4°C overnight, prior to incubation with horseradish peroxidase-conjugated secondary antibody goat anti-rabbit immunoglobulin G (IgG) (ab205718, 1:5,000; Abcam) for 2 h after the membrane was washed. Visualization of the protein bands was actualized using electrochemical luminescence, and scanning analysis was performed on a gel imager system. Thereafter, the gray level was determined by Image J software utilizing GAPDH as the internal control.

### 2.9 Cell counting kit-8 (CCK-8) assay

With the help of a CCK-8 kit (MedChemExpress, Monmouth Junction, NJ, USA), cell viability was tested. Briefly speaking, cells underwent digestion with 0.25% trypsin, and then inoculated onto 96-well plates (5 × 10^4^ cells/mL density) for 6 h, with three duplicates. At 0, 24, 48, and 72 h after cell attachment, 10 μL of CCK-8 solution was added to each well for 4 h cell culture. The absorbance value of the cells was estimated at 450 nm with an Envision Multilabel Reader (PerkinElmer, Waltham, MA, USA).

### 2.10 Flow cytometry assay

Cell apoptosis was tested utilizing an Annexin V-fluorescein isothiocyanate (FITC)/propidium iodide (PI) kit (CA1020, Beijing Solarbio Science & Technology Co., Ltd). The treated cells were collected and washed with PBS twice, ahead of 48 h incubation at 37°C with 5% CO_2_, followed by centrifugation and resuspension in 200 μL of binding buffer. The cells were added with 10 μL of Annexin V-FITC and 5 μL of PI for 15 min incubation in the dark at room temperature, followed by supplement with binding buffer (300 μL). At last, cell apoptosis at 488 nm excitation wavelength was investigated on a flow cytometer (e126037, B&D Biosciences, San Jose, CA, USA).

### 2.11 Measurement of mitochondrial membrane potential (Δ*Ψ*m)

Mitochondrial function in each group of cells was tested by evaluating intracellular levels of Δ*Ψ*m using fluorochrome tetramethylrhodamine methyl ester (TMRM; HY-D0984, MedChemExpress). A total of 5 μM TMRM was added into the culture medium, and the cells were cultured in the dark for 60 min and then washed three times. The fluorescence signals were observed and captured under a fluorescence microscope (XDS-500, Shanghai CAIKON Optical Instrument Co., Ltd, Shanghai, China).

### 2.12 Detection of ROS level

The ROS level was detected with 2, 7-dichlorodihydrofluorescein diacetate (DCFH-DA). According to the instructions of a ROS kit (HY-D0940, MedChemExpress), cells grown on the six-well plate were cultured as experimental design, and then the culture media in the plate were absorbed and discarded. Thereafter, 1 mL DCFH-DA with a final concentration of 10 μmol/L was added into each well, prior to incubation in the dark for 20 min (37°C), and the cells were washed with serum-free culture solution three times. Qualitative observation was performed using an inverted fluorescence microscope (XDS-500, Shanghai CAIKON Optical Instrument Co., Ltd), and quantitative detection of cell fluorescence intensity was implemented with a flow cytometry (e126037, BD Biosciences). The excitation wavelength and emission wavelength of DCFH-DA were 488 and 525 nm, respectively, and the intracellular ROS level was reflected by the fluorescence intensity.

### 2.13 Dual-luciferase reporter gene assay

The binding sites of miR-532-5p on ABL1 promoter were predicted via starBase database (https://starbase.sysu.edu.cn/). The wide-type sequence (wt-ABL1) and the mutant sequences (mut-ABL1) of the binding site were designed and synthesized separately according to the putative result and were inserted, respectively, into the luciferase reporter gene vector (pGL3-Promoter) which were then co-transfected with mimic-NC or miR-532-5p mimic into chondrocytes. After 48 h cell culture, Firefly and Renilla luciferase activities (transfected with phRL-TK vectors) were revealed with a dual-luciferase assay kit. Renilla luciferase activity acted as an internal control. The ratio of Firefly luciferase activity to Renilla luciferase activity was expressed as the relative luciferase activity.

### 2.14 RNA pull-down assay

The RNA pull-down assay was performed with a Pierce^TM^ Magnetic RNA-Protein Pull-Down kit (Millipore, MA, USA). In a nutshell, biotinylated miR-532-5p probes (Geneseed, Guangzhou, China) or biotinylated negative control (NC) probes were incubated with the lysates of chondrocytes at 25°C for 2 h. The miR-532-5p/ABL1 complex was captured by streptavidin-labeled immunomagnetic beads at 25°C for 1 h, prior to incubation with protease K-contained buffer at 25°C for another 1 h. The protein expression of ABL1 in the eluted complex was estimated by western blotting.

### 2.15 Statistical analysis

GraphPad Prism software (version 9.0) was applied to perform the statistical analysis. All obtained data were shown as mean ± standard deviation. The normality of continuous variables was assessed by the Shapiro–Wilk test. Under appropriate conditions, the chi-square test and/or unpaired student *t*-test were used to compare the demographic data between patients with nontraumatic ONFH and those with FNF. Data between two groups were compared with *t*-test, and one-way analysis of variance with Tukey’s multiple comparisons test was employed to compare data among multiple groups. Spearman correlation analysis was adopted for confirming the correlation between miR-532-5p and disease severity. The area under curve (AUC) of all tested markers was determined by receiver operating characteristic (ROC) curves. *P* value <0.05 was considered significantly different, and each test was repeated three times.

### 2.16 *Post hoc* statistical power calculation

The Power and Sample Size Calculators (http://powerandsamplesize.com) were used to calculate statistical power (1–*β*) in order to acquire data of different mean miR-532-5p levels, standard errors, as well as the number of registered patients in each group (https://doi.org/10.1201/9781584889830). Statistical power >0.8 was regarded strong, which was calculated as 1 – *β* = *φ*(*z* − *z*_1_ − *α*/2) + *φ* (−*z* − z_1_−*α*/2), *z* = (*μA* − *μB*)/*σ* (1/*n*_A_ + 1/*n*_B_).


**Ethical approval and informed consent:** Ethical approval was provided by the Ethics Committee of Tongji Hospital (No. YXLL-2023-262) and a written informed consent form was obtained from all participants allowing the use of their blood samples and femoral head cartilage specimens. This study was conducted in accordance with the Declaration of Helsinki.

## 3 Results

### 3.1 miR-532-5p was downregulated in patients with nontraumatic ONFH

**Figure 1:**
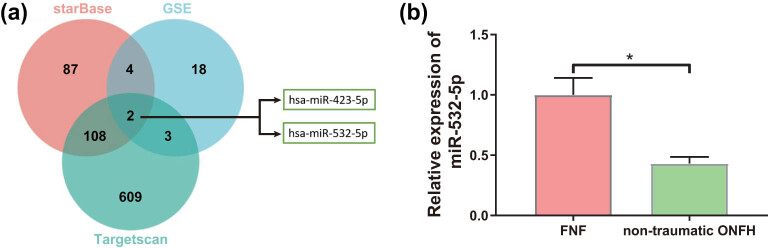
Downregulation of miR-532-5p in patients with nontraumatic ONFH. (a) Venn analysis of miRNAs targeting to ABL1 and (b) the expression of miR-532-5p in tissues of nontraumatic ONFH and FNF patients was measured by qRT-PCR assay. *P < 0.05. *T*-test was utilized for the comparison between two groups, and all experiments were repeated three times.

Based on a previous analysis of GSE74089 and GO database, the top ten oxidative stress-related hub genes in ONFH were PTGS2, JUN, CASP3, RELA, ABL1, JAK2, FBXW7, FOXO3, MCL1, EZH2, among which RELA was downregulated and the other nine genes were upregulated in ONFH [15]. Wang et al. screened out 27 ONFH-specific serum miRs by deep sequencing technology, among which 15 miRs including miR-423-5p and miR-3960 were significantly upregulated compared with healthy controls, while another 12 microRNAs including miR-100-5p, miR-99a-5p, and miR-532-5p were downregulated in ONFH [23]. Furthermore, a Venn diagram of the above 27 miRs and the miRs targeting ABL1 in Starbase (https://starbase.sysu.edu.cn/index.php) and Targetscan databases (https://www.targetscan.org/vert_71/) was plotted (Tables S1 and S2), whose results manifested that only hsa-miR-423-5p and hsa-miR-532-5p overlapped in the three databases (Figure 1a). Previous studies indicated that ABL1 was overexpressed in ONFH, prompting us to identify whether miR-532-5p could affect the occurrence and development of ONFH by targeting ABL1.

In this work, 96 patients (53 males and 43 females) with nontraumatic ONFH were enrolled at an average age of 52.19 ± 8.96 years. In parallel, 96 patients with FNF, including 50 males and 46 females, were selected as controls with an average age of 50.76 ± 7.23 years. There were no obvious differences in age, sex, and BMI between nontraumatic ONFH patients and FNF patients (control group) (Table 2, *P* > 0.05). After calculation, the statistical power was 0.89, showing that the sample size of our study was enough to draw conclusions.

**Table 2:** Demographic data of enrolled participants

Clinicopathologic features	Nontraumatic ONFH patients (*n* = 96)	FNF patients (*n* = 96)	*P* value
Age (years)	52.19 ± 8.96	50.76 ± 7.23	0.225
Sex (F/M)	43/53	46/50	0.772
BMI (kg/m^2^)	23.5 ± 2.38	23.1 ± 2.33	0.241
ARCO stage (2/3/4)	31/33/32	/	
VAS score	6.5 ± 0.81	/	
HSS score	67.9 ± 7.25	/	
Serum miR-532-5p level	0.56 ± 0.12	1 ± 0.21	<0.001
Serum LDH level (U/L)	179.35 ± 15.27	82.76 ± 10.16	<0.001
Serum SOD level (U/mL)	12.26 ± 3.36	37.29 ± 4.13	<0.001
Serum MDA level (mmol/mL)	36.08 ± 3.79	10.17 ± 2.32	<0.001

Notes: All data given as mean value ± SD or median; *P* < 0.05 is considered as significant difference.

Moreover, qRT-PCR assay displayed that miR-532-5p expression was notably reduced in tissues of nontraumatic ONFH patients compared to that of the controls (Figure 1b, *P* < 0.05), and miR-532-5p expression in serum of nontraumatic ONFH patients was also decreased (Table 2, *P* < 0.05), suggesting a crucial role of miR-532-5p in nontraumatic ONFH. At the same time, results of ELISA revealed that LDH and MDA were dramatically increased, while SOD was noticeably decreased in serum of nontraumatic ONFH patients (Table 2, *P* < 0.05). Hence, we surmised that miR-532-5p may be involved in the occurrence and development of nontraumatic ONFH through influencing oxidative stress in patients with nontraumatic ONFH.

### 3.2 Reduced miR-532-5p may serve as a potential marker of radiological progression in nontraumatic ONFH

**Figure 2:**
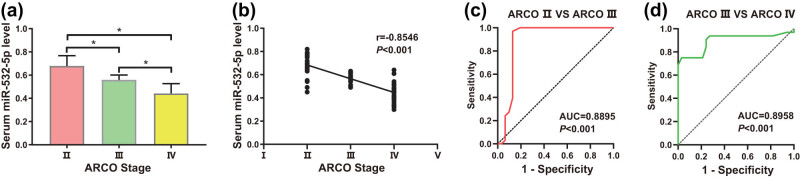
Downregulated miR-532-5p may function as a possible marker of radiological progression in nontraumatic ONFH. (a) qRT-PCR assay was used to investigate the level of miR-532-5p in nontraumatic ONFH patients with different stages of ARCO, (b) correlation analysis between miR-532-5p level in serum and ARCO staging, (c) ROC curve analysis of miR-532-5p level in patients with ARCO stage II and patients with ARCO stage III, and (d) ROC curve analysis of miR-532-5p level in patients with ARCO stage III and patients with ARCO stage IV. Differences were considered significant at *P* < 0.05.

Detection of miR-532-5p levels in 96 nontraumatic ONFH patients with different ARCO stages was implemented. After screening, patients with nontraumatic ONFH were divided into three groups according to the ARCO staging system. The nontraumatic ONFH group consisted of 31 patients with ARCO stage II, 33 patients with ARCO stage III, and 32 patients with ARCO stage IV. In comparison with nontraumatic ONFH patients in ARCO stage III and II, miR-532-5p in serum was obviously lessened in patients with ARCO stage IV, and its expression was significantly lower in patients with ARCO stage III than in patients with ARCO stage II (Figure 2a, *P* < 0.05). miR-532-5p expression was negatively associated with ARCO staging (*r* = −0.8546, *P* < 0.001) (Figure 2b).

ROC curve analysis was conducted to explore the diagnostic value of miR-532-5p for radiographic progression. For both ARCO stage II vs III and III vs IV, downregulated miR-532-5p demonstrated significant AUC (AUC = 0.8895, *P* < 0.001; AUC = 0.8958, *P* < 0.001) (Figure 2c and d, *P* < 0.05). Those findings illustrated that decreased miR-532-5p may serve as a potential marker of radiographic progression at all stages of nontraumatic ONFH.

### 3.3 Serum miR-532-5p was negatively correlated with VAS, LDH, and MDA and positively related to HHS and SOD

**Figure 3:**
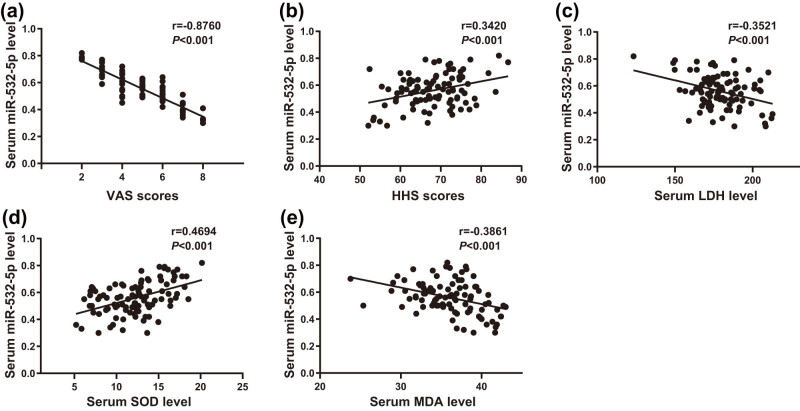
miR-532-5p was in negative relation with VAS, LDH, and MDA, and positive association with HHS and SOD. (a) Correlation analysis between the level of miR-532-5p in serum and VAS, (b) correlation analysis between the level of miR-532-5p in serum and HHS, and (c)–(e) analysis on the correlation between the expression level of miR-532-5p in serum and oxidative stress-related indexes (LDH, SOD, and MDA). Differences were considered significant at *P* < 0.05.

VAS and HHS scores unveiled that the expression level of miR-532-5p was negatively related to VAS (*r* = −0.8760, *P* < 0.001) (Figure 3a), and positively associated with HHS (*r* = 0.3420, *P* < 0.001) (Figure 3b). In addition, the level of miR-532-5p in serum was found to be inversely relevant with serum LDH and MDA levels (*r* = −0.3521, *P* < 0.001; *r* = −0.3861, *P* < 0.001) (Figure 3c and e), and was positively correlated with serum SOD level (*r* = 0.4694, *P* < 0.001) (Figure 3d).

### 3.4 Overexpressed miR-532-5p alleviated chondrocyte damage and oxidative stress caused by nontraumatic ONFH

**Figure 4:**
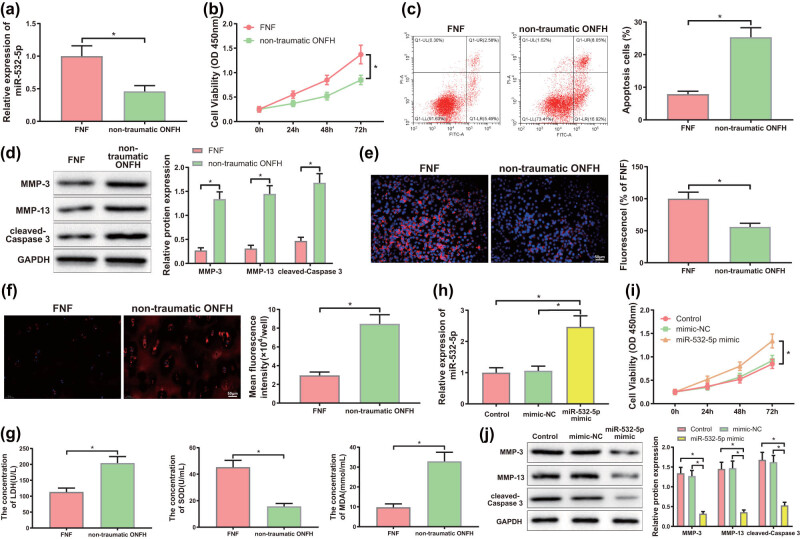

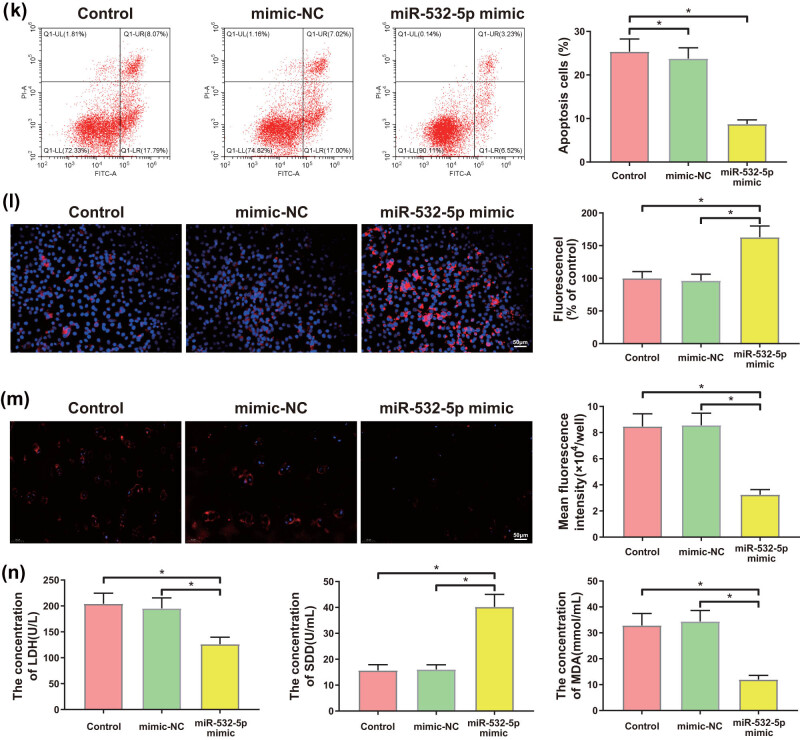
Overexpression of miR-532-5p relieved chondrocyte damage and oxidative stress caused by nontraumatic ONFH. (a) and (h) Expression of miR-532-5p in chondrocytes of each group was examined by qRT-PCR assay, (b) and (i) cell viability of chondrocytes in each group was probed by CCK-8 assay, (c) and (j) cell apoptosis of chondrocytes in each group was measured using flow cytometry, (d) and (k) western blotting was utilized to investigate expression of MMP-3, MMP-13, and cleaved-caspase3 in each group of chondrocytes, (e) and (l) fluorescence microscopy was adopted for observation of changes of mitochondrial membrane potential (Δ*Ψ*m) in chondrocytes, (f) and (m) the ROS level in two groups of chondrocytes was tested by DCFH-DA, (g) and (n) ELISA was used to determine the levels of LDH, SOD, and MDA in each group.*P < 0.05. *T*-test was implemented for the comparison between two groups. One-way analysis of variance was employed to compare data among multiple groups, and corrected by Tukey’s multiple comparisons test. All experiments were repeated for three times.

To further pinpoint the effect of miR-532-5p in ONFH, we extracted chondrocytes from cartilage tissues of femoral head of patients with nontraumatic ONFH and patients with FHF. Results of qRT-PCR assay showed that the level of miR-532-5p was distinctly reduced in cartilage tissues of femoral head of nontraumatic ONFH patients versus FHF patients (Figure 4a, *P* < 0.05), which was consistent with the expression pattern of miR-532-5p in serum of the two groups. CCK-8 and flow cytometry assays enunciated that cell viability in chondrocytes isolated from cartilage tissues was evidently weaker and cell apoptosis rate was strengthened in nontraumatic ONFH group than in FNF group (Figure 4b and c, *P* < 0.05). Western blotting displayed that the expression of MMP-3, MMP-13, and cleaved-caspase3 was clearly increased in chondrocytes of nontraumatic ONFH group versus FNF group (Figure 4d, *P* < 0.05). Besides, Δ*Ψ* in nontraumatic ONFH group was observed by a fluorescence microscope and the results revealed remarkably lower Δ*Ψ* in nontraumatic ONFH group than in FNF group (Figure 4e, *P* < 0.05), but ROS level was conspicuously enhanced in nontraumatic ONFH group than in FNF group (Figure 4f, *P* < 0.05). ELISA elucidated notable increases in LDH and MDA, and a marked decline in SOD content in nontraumatic ONFH group compared to those in FNF group (Figure 4g, *P* < 0.05).

Afterward, mimic-NC and miR-532-5p mimic were transfected into chondrocytes of nontraumatic ONFH group, which were next categorized into control group (without transfection), mimic-NC group, and miR-532-5p mimic group. qRT-PCR elucidated that the expression of miR-532-5p was strikingly higher in miR-532-5p mimic group (vs control group and mimic-NC group), stating a successful transfection (Figure 4h, *P* < 0.05). CCK-8 and flow cytometry assays revealed enhanced cell viability and inhibited cell apoptosis in miR-532-5p-overexpressing chondrocytes (Figure 4i and j, *P* < 0.05). Western blotting exhibited obviously restrained expression of MMP-3, MMP-13, and cleaved-caspase3 in miR-532-5p mimic group compared with that in mimic NC group (Figure 4k, *P* < 0.05). A significant increase in Δ*Ψ* (Figure 4l, *P* < 0.05) and a distinct decline in ROS (Figure 4m, *P* < 0.05) were revealed in miR-532-5p mimic group compared with that in mimic NC group. ELISA indicated that the levels of LDH and MDA were notably diminished but SOD level dramatically rose in miR-532-5p-overexpressing chondrocytes (Figure 4n, *P* < 0.05). Taken together, miR-532-5p overexpression played an alleviative role in chondrocyte damage and oxidative stress induced by nontraumatic ONFH.

### 3.5 miR-532-5p downregulated ABL1 expression in a targeted fashion

**Figure 5:**
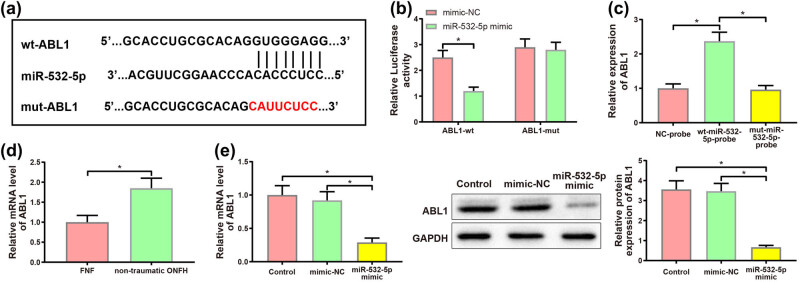
miR-532-5p downregulated the expression of ABL1 in a targeted manner. (a) StarBase database predicted the direct binding site of miR-532-5p to ABL1, and the wt-ABL1 and mut-ABL1 on ABL1 were designed, (b) the binding of miR-532-5p and ABL1 was surveyed by dual-luciferase reporter gene assay, (c) RNA pulldown verified the interaction between miR-532-5p and ABL1, (d) qRT-PCR was used to determine the expression of ABL1 mRNA in cartilage tissues of patients with nontraumatic ONFH and patients with FNF, and (e) ABL1 expression in each group of chondrocytes was investigated by qRT-PCR and western blotting. *P < 0.05. *T*-test was implemented for the comparison between two groups. One-way analysis of variance was employed to compare data among multiple groups, and corrected by Tukey’s multiple comparisons test. All experiments were performed in triplicate.

Direct binding site of miR-532-5p to ABL1 was predicted via starBase database. The wt-ABL1 and mut-ABL1 of the binding site were designed and synthesized (Figure 5a), and subsequently inserted into the luciferase reporter gene vector. Results of dual-luciferase reporter gene assay illustrated that luciferase activity was remarkably lower in chondrocytes co-transfected with the wt-ABL1 vector and miR-532-5p mimic in comparison with the chondrocytes co-transfected with wt-ABL1vector and mimic-NC (Figure 5b, *P* < 0.05). However, there was no noticeable difference in the luciferase activity in chondrocytes transfected with the mut-ABL1 vector after transduction with miR-532-5p mimic and mimic-NC. RNA pull-down assay displayed that miR-532-5p probes conspicuously enriched ABL1 (Figure 5c, *P* < 0.05). Altogether, miR-532-5p targeted ABL1.

Next, qRT-PCR assay showed that ABL1 mRNA was markedly strengthened in cartilage tissues of nontraumatic ONFH patients versus FNF patients (Figure 5d, *P* < 0.05). Next, qRT-PCR and western blotting demonstrated that ABL1 expression was notably reduced in miR-532-5p mimic group compared with that in mimic-NC group (Figure 5e, *P* < 0.05). These results hinted that miR-532-5p may mitigate the chondrocyte damage caused by nontraumatic ONFH by suppressing ABL1.

### 3.6 Upregulated ABL1 partially reversed the mitigating effects of miR-532-5p upregulation on nontraumatic ONFH-induced chondrocyte damage and oxidative stress

**Figure 6:**
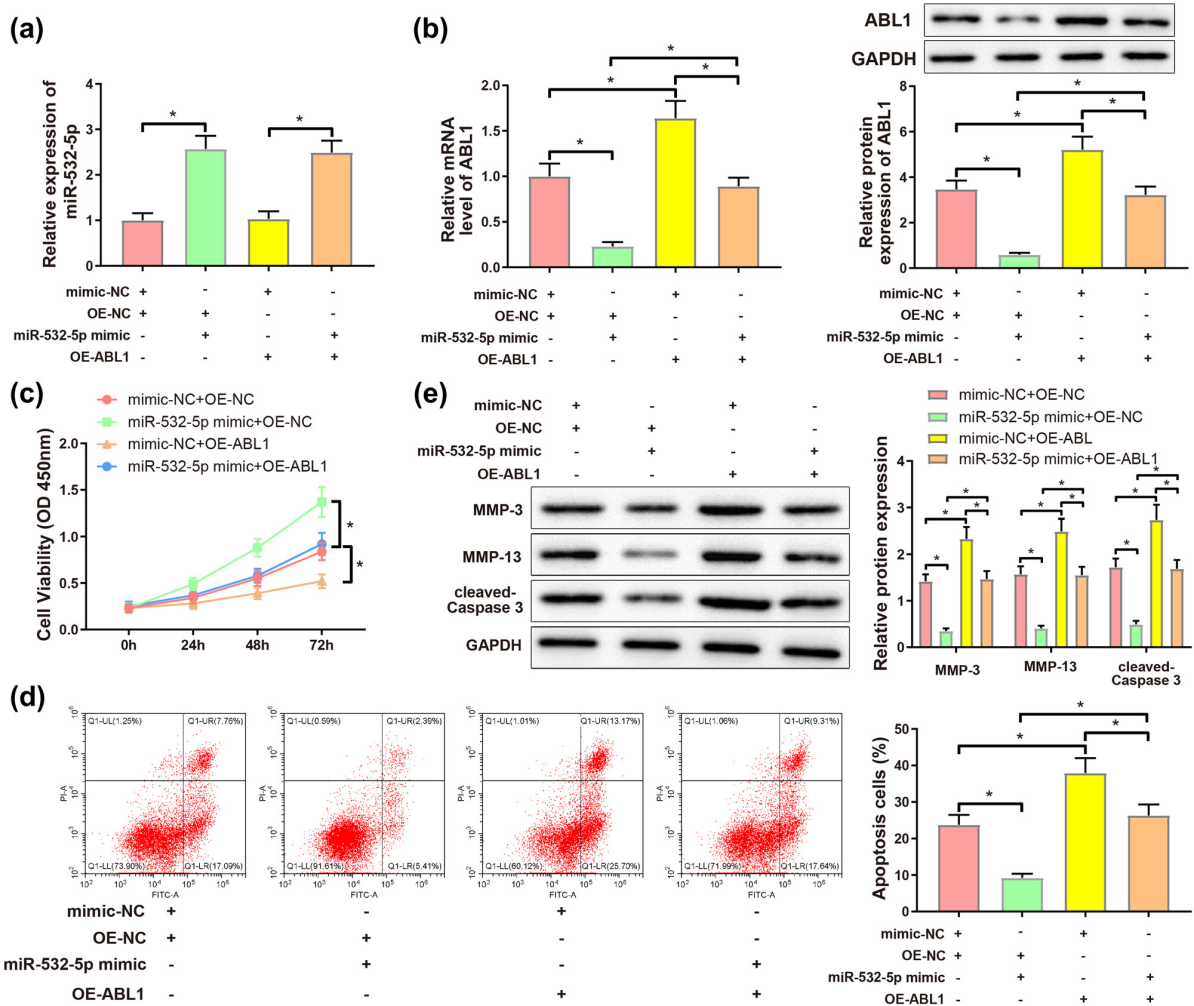

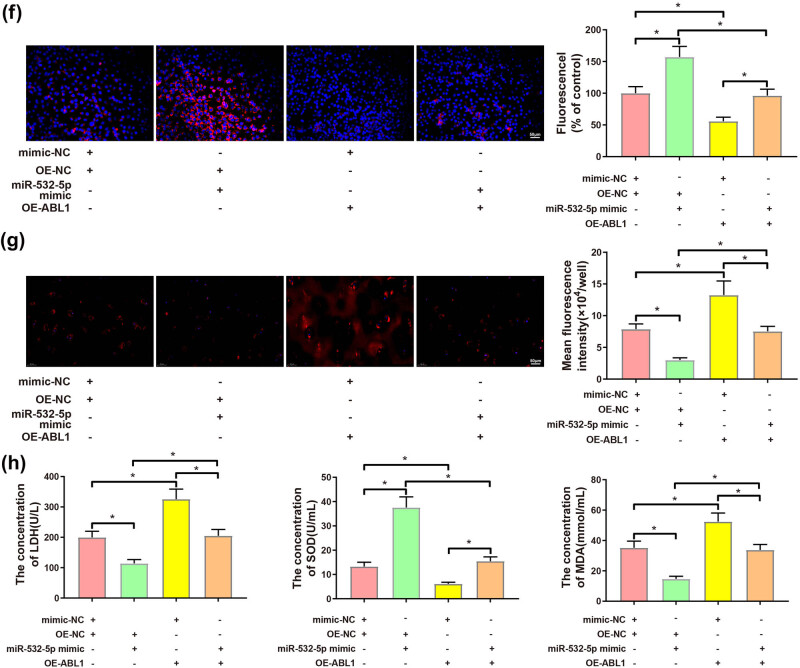
ABL1 upregulation partially reversed the beneficial impact on chondrocytes induced by miR-532-5p overexpression. (a) Expression of miR-532-5p in each group of chondrocytes was determined by qRT-PCR, (b) ABL1 in chondrocytes of each group was measured with qRT-PCR and western blotting assays; (c) CCK-8 assay was employed to detect cell viability, (d) cell apoptosis was investigated by flow cytometry, (e) western blotting demonstrated the expression of MMP-3, MMP-13, and cleaved-caspase3, (f) changes of Δ*Ψ* were observed under a fluorescence microscope, (g) the levels of ROS in chondrocytes were determined by DCFH-DA, and (h) ELISA displayed the levels of LDH, SOD, and MDA. *P < 0.05. One-way analysis of variance was employed to compare data among multiple groups, and corrected by Tukey’s multiple comparisons test. All experiments were performed in triplicate.

The chondrocytes isolated from cartilage tissues of nontraumatic ONFH patients were divided into mimic-NC + OE-NC, miR-532-5p mimic + OE-NC, mimic-NC + OE-ABL1, and miR-532-5p mimic + OE-ABL1 groups. As revealed by qRT-PCR and western blotting assays, miR-532-5p expression was significantly enhanced, while ABL1 expression was dramatically decreased in miR-532-5p mimic + OE-NC group versus mimic-NC + OE-NC group; the expression of miR-532-5p was obviously higher in miR-532-5p mimic + OE-ABL1 group than in mimic-NC + OE-ABL1 group. Meanwhile, ABL1 expression in miR-532-5p mimic + OE-ABL1 group was increased compared to that in miR-532-5p mimic + OE-NC group, and noticeably lessened versus mimic-NC + OE-ABL1 group (Figure 6a and b, *P* < 0.05). CCK-8 assay enunciated that cell viability was distinctly higher in miR-532-5p mimic + OE-NC group than in mimic-NC + OE-NC group and miR-532-5p mimic + OE-ABL1 group. Additionally, cell viability in miR-532-5p mimic + OE-ABL1 group was strikingly increased in comparison with mimic-NC + OE-ABL1 group (Figure 6c, *P* < 0.05). However, cell apoptosis was repressed in miR-532-5p mimic + OE-NC group than in mimic-NC + OE-NC and miR-532-5p mimic + OE-ABL1 groups, and miR-532-5p mimic + OE-ABL1 group had decreased apoptosis rate compared with mimic-NC + OE-ABL1 group (Figure 6d, *P* < 0.05). Western blotting displayed that the expression of MMP-3, MMP-13, and cleaved-caspase3 was evidently weakened in miR-532-5p mimic + OE-NC group (vs mimic-NC + OE-NC and miR-532-5p mimic + OE-ABL1 groups), while these proteins were remarkably inhibited in miR-532-5p mimic + OE-ABL1 group than in mimic-NC + OE-ABL1 group (Figure 6e, *P* < 0.05). Observation under the fluorescence microscope showed that Δ*Ψ* of chondrocytes was strikingly higher in miR-532-5p mimic + OE-NC group compared to that in mimic-NC + OE-NC and miR-532-5p mimic + OE-ABL1 groups, and significantly increased in miR-532-5p mimic + OE-ABL1 group versus mimic-NC + OE-ABL1 group (Figure 6f, *P* < 0.05). The ROS level was downregulated in miR-532-5p mimic + OE-NC group than that in mimic-NC + OE-NC and miR-532-5p mimic + OE-ABL1 groups; compared with mimic-NC + OE-ABL1 group, miR-532-5p mimic + OE-ABL1 group had lower ROS level (Figure 6g, *P* < 0.05). ELISA illustrated that compared with those in mimic-NC + OE-NC group, the levels of LDH and MDA were significantly decreased while the level of SOD was markedly increased in miR-532-5p mimic + OE-NC group. The LDH and MDA levels in miR-532-5p mimic + OE-ABL1 group were notably higher than those in miR-532-5p mimic + OE-NC group, and obviously lower than those in mimic-NC + OE-ABL1 group. SOD level in miR-532-5p mimic + OE-ABL1 group was decreased than in miR-532-5p mimic + OE-NC group and increased compared with that in mimic-NC + OE-ABL1 group (Figure 6h, *P* < 0.05). Above findings illustrated that ABL1 upregulation partially reversed the alleviating effect of miR-532-5p overexpression on chondrocyte injury and oxidative stress induced by nontraumatic ONFH, without affecting the expression of miR-532-5p.

## 4 Discussion

Nontraumatic ONFH frequently occurs in adults younger than 50 years, resulting in femoral head collapse and arthritis of the hip [24]. Alleviating joint burden and application of nonsteroidal drugs are utilized for early ONFH [25]. For advanced patients with ONFH, total hip arthroplasty (THA) is the most effective treatment [26]. However, the outcomes of THA treatment in young patients or in the active population are often unsatisfactory, and accompanied by a number of prosthesis-related complications [27], leaving it urgent to seek more effective and safe treatments for ONFH. The current study aims to investigate the role of miR-532-5p in oxidative stress response and the significance of serum miR-532-5p in the diagnosis of nontraumatic ONFH. Experimental results illustrated that serum miR-532-5p had a diagnostic value in nontraumatic ONFH, and ameliorated nontraumatic ONFH-induced oxidative stress in chondrocytes by suppression of ABL1.

miR-532-5p is located on Xp11.23 of the human chromosome [28]. Its expression has been found to be increased in differentiated chondrocytes than in precursor chondrocytes from human embryonic cartilage tissues [29]. Upregulated miR-532-5p increases the cell viability and colony formation of HT-29 cells via targeting of the 5 ʹ-UTR of RUNX3 [30]. Recently, miR-532-5p was predicted to be upregulated in traumatic ONFH [31]. In sepsis-related nontraumatic ONFH, miR-532-5p was found to be under-expressed [23]. In the present study, miR-532-5p was demonstrated to be downregulated in tissues and serum of nontraumatic ONFH patients. Combined with the previous findings, we found that miR-532-5p may have different expression patterns in traumatic and nontraumatic ONFH. Moreover, miR-532-5p was indicated to be a potential prognosis biomarker for patients with osteosarcoma [32]. Herein, the serum level of miR-532-5p was negatively related with ARCO staging, which suggested that miR-532-5p downregulation may serve as a potential marker of radiological progression in all stages of nontraumatic ONFH. Additionally, ARCO staging is an improvement on Ficat-Arlet staging and Steinberg staging, which can be combined with radiographs, CT, MRI, and perfusion imaging to determine the location and size of the necrotic area [21]. The advantage of ARCO staging is that it is more concise and practical, but its disadvantage is that the efficiency of collapse prediction and treatment guidance may be affected. Besides, the potential relationship between serum miR-532-5p expression levels and symptom severity was identified using the VAS and HHS scores. In parallel, changes of oxidative stress indexes were found in the serum of nontraumatic ONFH. Several clinical disorders including vascular damage and cell apoptosis are linked to oxidative stress, which is in intimate connection with ONFH [33]. These findings prompted us to hypothesize that miR-532-5p may act a role in the occurrence and development of nontraumatic ONFH by influencing the oxidative stress level.

Radiographic changes can be seen in the early stages of ONFH, including roughness of the cartilage surfaces [34], indicating the crucial role of cartilage in the development of ONFH. Chondrocytes have different responses to growth stimulants depending on their intracellular redox state, and ROS acts as a key factor that affects intracellular redox status [35]. In nontraumatic ONFH, overexpressed miR-532-5p decreased the levels of LDH and MDA, and enhanced the level of SOD and cell viability of chondrocytes. Therefore, miR-532-5p overexpression alleviated nontraumatic ONFH-caused chondrocyte damage and oxidative stress and elevated Δ*Ψ*m level in chondrocytes. These changes in chondrocyte biological function ultimately enhanced the activity of chondrocytes and reduce the level of apoptosis. Finally, the development of nontraumatic ONFH can be alleviated by promoting the growth of chondrocytes, so as to treat nontraumatic ONFH patients. Reportedly, ABL1 is involved in mitochondrial activity, ATP metabolism, protein translation and metabolism, and numerous neurological disorders [36]. Growth factors, adhesion receptors, chemokines, oxidative stress, and DNA damage are among the stimuli that activate ABL1 [37]. In the present work, analysis from starBase database predicted that miR-532-5p directly bound to ABL1. A recent report has revealed that inhibition of the ABL kinases diminished tumor outgrowth and impaired metastatic spread [38]. ABL1 was highly expressed in nontraumatic ONFH, and RNA pull-down assay illustrated that miR-532-5p probes dramatically enriched ABL1. Further, miR-532-5p inhibited ABL1 in a targeted fashion to alleviate nontraumatic ONFH-induced chondrocyte damage by affecting the levels of ROS, LDH, MDA, and SOD, the change of Δ*Ψ*m level, and oxidative stress degree. Moreover, experimental results displayed that overexpression of ABL1 partially reversed the mitigating effect of miR-532-5p on nontraumatic ONFH-induced chondrocyte damage and oxidative stress without affecting miR-532-5p expression.

## 5 Limitations

However, there are also some limitations in our study. First, through research review, the pathological mechanism of ONFH is complex. In addition to oxidative stress in this study, we can conduct related studies from more aspects in the future, such as cell apoptosis, angiogenesis, metastasis, and stem cell differentiation. Second, our findings remain to be translated from animal models into clinical settings. In the future, we will explore the mechanism of miR-532-5p/ABL1 in several modes of cell death, so as to provide more theoretical knowledge for clinical treatment of ONFH.

## 6 Conclusion

Overall, our discoveries illustrated that miR-532-5p suppressed ABL1 expression in a targeted manner to reduce chondrocyte damage and oxidative stress caused by nontraumatic ONFH. Moreover, we identified the negative correlation between miR-532-5p and radiographic progression and symptomatic severity, which indicated that miR-532-5p may have a diagnostic value in nontraumatic ONFH. This study may provide a clue for further exploring the pharmacological approaches for treating nontraumatic ONFH. Our study is similar to previous studies in this field in that it is from the perspective of non-coding RNA, so as to explore the direct downstream target of miRNA and influence the biological activity of chondrocytes through the expression of related proteins. However, the difference was that we first screened the most differentially expressed miRNAs in nontraumatic ONFH through bioinformatics analysis, and then clinically verified the relationship between the expression level of miR-532-5p in serum of patients and imaging severity and biochemical indicators. After we know these results, we can proceed with cell validation experiments. This will help readers to explore the potential mechanism of miRNA therapy for nontraumatic ONFH from both clinical and cellular perspectives.

## Supplementary Material

Supplementary Table 1

Supplementary Table 2
